# Healthy pregnancies and essential fats: focus group discussions with Zambian women on dietary need and acceptability of a novel RUSF containing fish oil DHA

**DOI:** 10.1186/s12884-020-2783-8

**Published:** 2020-02-10

**Authors:** Catherine Chunda-Liyoka, Mwansa Ketty Lubeya, Mercy Imakando, Sophia Kisling, Sonoor Majid, Mary S. Willis, Charles Wood, Chipepo Kankasa, Concetta C. DiRusso

**Affiliations:** 1 University Teaching Hospitals, Lusaka Children’s Hospital, University of Zambia, School of Medicine, Lusaka, Zambia; 2 University Teaching Hospitals, Women and Newborn Hospital, University of Zambia, School of Medicine, Lusaka, Zambia; 3 Nebraska Center for Integrated Biomolecular Communications, Department of Biochemistry, N241 Beadle Center, University of Nebraska-Lincoln, Lincoln, NE 68588 USA; 4 Department of Nutrition and Health Sciences, University of Nebraska-Lincoln, Lincoln, NE USA; 5 Nebraska Center for Virology, School of Biological Sciences, University of Nebraska-Lincoln, Lincoln, NE USA

**Keywords:** Ready to use supplemental foods (RUSF), Docosahexaenoic acid (DHA), Omega 3, Focus groups, Hedonic assessment, Malnutrition, Food insecurity, Zambia, Pregnancy

## Abstract

**Background:**

Nut butter-based Ready to Use Supplemental Foods (RUSF) are an effective way to add nutrients and calories to diets of malnourished and food insecure populations. The RUSF formulations have been further modified to add micronutrients including iron and folic acid needed during pregnancy and lactation. Because docosahexaenoic acid (DHA, C22:6 n-3) enhances fetal development and birth outcomes, it has been suggested that perhaps RUSF formulations for pregnancy should also include this Omega 3 fatty acid. The goal of the present study was to gain an understanding of Zambian women’s knowledge of nutritional needs in pregnancy through structured focus group discussions, and to formulate and determine the acceptability of a RUSF with DHA.

**Methods:**

Structured focus group sessions were conducted among women attending an antenatal clinic at the University Teaching Hospitals in Lusaka, Zambia. Dietary and nutrition knowledge was surveyed through structured dialogue that was recorded by audio and transcribed verbatim. An RUSF containing 400 mg DHA from fish oil in 50 g RUSF was designed and assessed for fatty acid content and product stability. Participants then sampled the RUSF-DHA, provided feedback on taste, and were surveyed about willingness to consume the novel formula using a standardized hedonic instrument.

**Results:**

The participants’ knowledge of foods recommended for use in pregnancy included fruits, vegetables, meat, and fish. Most women reported eating fish at least once per week, although the specific type of fish varied. Most did not have prior knowledge of the importance of consuming fish during pregnancy or that some fish types were more nutritional than others as they included omega 3 fatty acids. The participants were uniformly accepting of the RUSF-DHA for the purpose of enhancing birth and developmental outcomes, but were critical of the aroma in hedonic testing.

**Conclusions:**

Women were committed to consuming a healthy diet that would impact the outcome of pregnancy, and were receptive to advice on the importance of consuming foods such as fish as a source of DHA. The RUSF-DHA formulation was acceptable due to the potential benefits for the developing infant, however, the fishy odor may be limiting for long-term daily use.

## Background

Maternal, infant, and child nutrition is accepted as a key determinant of health that is vital to human capital development and, as such, is a high priority for Sub-Saharan Africa [1, 2]. Zambia has a population of approximately 16 million and about half of these are women of reproductive age (15–49) [1]. More than a third of women give birth to their first child before age 18 and 50% by the age of 20. Additionally, 10% of women aged 15–49 are underweight (BMI < 18.5 kg/m^2^),) and 14.3% of women in Zambia are living with HIV. Low pre-pregnancy BMI, short stature and HIV are among the risk factors for poor birth outcomes. In the under-five age group, 14.8% are underweight and 40% are stunted, with 17% severely stunted. It is estimated that only 11% of children aged 6–23 months are fed adequately based upon recommended feeding practices [1]. About 72,000 children (0–14 years) are infected with HIV. Due to the high rates of both malnutrition and HIV infection in Zambia, it is essential that nutrition be optimized to maximize development, growth, and the ability to resist and fight infectious disease [2, 3].

Improving maternal and infant nutrition and health is a major focus of the Zambian Government to ensure the population reaches its full potential [1, 4]. However, the dietary diversity for women and children in Zambia is highly dependent upon socioeconomic status, such that a low diversity index correlates with malnutrition and food insufficiency [5, 6]. Animal-source foods are limited and the diet is highly dependent on starchy staples such as the corn-based porridge, *nshima*. Therefore, the basic diet of the very low income population has limited diversity, contributing to insufficient daily protein and micronutrient intake, and minimal consumption of long chain polyunsaturated n-3 fatty acids [1]. Fish is an important staple in the Zambian diet and fish intake correlates with dietary diversity and lower levels of stunting [2, 5, 7]. The small sardine-like fish species, *kapenta*, are a rich source of DHA and native to Zambia [7]. However, in recent years, fish has become expensive and availability more limited, especially in urban Zambia where the bulk of the population resides.

In cases of undernutrition, intervention and recovery programs can help to improve nutritional status. These often include Ready to Use Therapeutic Foods (RUTF) that are formulated to treat moderate (MAM) and severe acute malnutrition (SAM) [8, 9]. The base of one class called lipid nutritional supplements (LNS) is nut paste, generally peanut butter, that is supplemented to meet daily macro- and micro-nutrient requirements including the essential fatty acids [8, 10, 11]. When first developed, the sole purpose of these lipid based RUTFs was short-term use in SAM to raise the individual’s body weight over 3–4 weeks after which the patient could be transitioned to a conventional diet [10]. In actuality, due to pervasive malnutrition and food insecurity, the therapeutic foods are used over months to supplement the diet of children with MAM and SAM under the age of five and pregnant or lactating women [11, 12]. This has led to the development of Ready to Use Supplemental Foods (RUSF) that provide the daily requirement for essential micronutrients, and a lesser amount of the macronutrients, to bring added value to the daily food intake and enhance, but not replace, a conventional diet.

One point of concern with the lipid-based RUTFs is that the essential polyunsaturated fatty acids in these food supplements are from plant oils and include only linoleic acid (LA n-6) and linolenic acid (LN n-3). These two fatty acids must be further processed in the body to make the long chain polyunsaturated fatty acids arachidonic acid (AA n-6), eicosapentaenoic acid (EPA n-3) and docosahexaenoic acid (DHA n-3). This biological process is inefficient in humans [13]. Because DHA is a building block for fetal brain growth, large amounts are required during fetal development and for 24 months after birth [14]. When a pregnant woman is food insecure, her fetus, and later her infant, are at risk for suboptimal brain development and function. If both food insecure and living with HIV, fetal brain development and function are further at risk [2, 15, 16]. Therefore, there is much interest in developing dietary supplements that include DHA, during pregnancy and lactation, to support neuronal development [17, 18].

Given the high rate of malnutrition and stunting in Zambia and the value of RUSF for use in addressing some of the nutritional problems of women and their infants, we developed an RUSF with fish oil to supply the DHA crucial to fetal and infant brain growth. The goals of the present study were to assess the knowledge of pregnant women about what foods are required during pregnancy for maximizing fetal development and, more specifically, to address knowledge about the need for essential fatty acids including DHA in the diet for brain development. Employing focus group discussions surrounding nutrition and health, as well as hedonic testing of the RUSF-DHA, the current study presents findings on nutrition knowledge for pregnancy and the acceptability the peanut butter-based RUSF with fish oil to pregnant women in Zambia.

## Methods

### Study site

This work was a collaboration between the University Teaching Hospitals (UTHs), Lusaka, Zambia and the University of Nebraska, Lincoln, Nebraska, U.S.A. Experimental design, ethical conduct of the research, and the data sharing plan were approved by Institutional Review Boards at each institution to comply with Zambian and US laws (UNZAREC IRB #00005948 and UNL IRB #20170416833FB). Experimental design met NIH policies regarding conflict of interest, biosafety, human participant research, data collection and analysis, and confidentiality.

This study took place in Lusaka, Zambia, at the UTHs, which are part of a complex of hospitals that include the Women and Newborn Hospital, Children’s Hospital, Adult and Emergency Hospital, Eye Hospital and Cancer Diseases Hospital. Lusaka is the largest city in Zambia with a population of about 2.4 million inclusive of the metropolitan area, which is 15% of the total national population. The UTHs comprise the largest health care center in the country. The Women and New Born Hospital of the UTHs facilitate approximately 60–80 antenatal clinic screenings performed per day and 900–1000 deliveries per month, on average, providing a large patient population pool for the studies reported here.

### Participants

All participants were recruited from the antenatal clinic of the Women and Newborn Hospital in Lusaka. The individuals were selected for participation in the focus group at random from a pool of approximately 150 women being recruited for a larger preclinical trial. The participant pool was made up of women of reproductive age who were pregnant and of a typical pre-pregnancy body mass, i.e., between 18 and 25 kg/m^2^ and neither over- or under-weight. Referral for participation was made through collaborating medical doctors in the UTH Women and Newborn Hospital antenatal care clinic (ANC). Information about the study was verbally given to potential participants by study nurses and/or doctors. Those that understood and accepted the invitation were asked to provide written informed consent to be included in the study.

### Focus group site and procedures

The sessions were held in the ANC meeting and training room. The room was an open space with rows of benches used for antenatal lessons and clinic appointment registration holding about 60–80 persons at capacity. The focus groups were held at times when the room was not scheduled for other purposes. However, the outside entrance was at the back of the room, consequently, some individuals who were not participating could enter or exit during focus group sessions. The number of non-participants never exceeded 5–10 and these individuals stayed at the back of the room away from the discussions. Non-participants were instructed to remain silent during session conversations.

Three focus group sessions with 7–10 participants (totaling 27) were held on 3 different days between September and November 2017. This number was considered adequate to engage participants in a conversation without inhibiting more reticent individuals [19]. A core team of professionals ran the sessions and included two physicians (authors MKL and MI), one study nurse and one nutritionist. All had experience in conducting sessions of this type for the purpose of teaching and research. Only the nutritionist was male, and although session leadership roles varied, the same professionals participated in all 3 sessions using the same script. The predominant language used during focus groups was *Nyanja* with some English, which was understood by most participants. In one of the groups, there was a client who could not understand *Nyanja*/English, so *Bemba* had to be used as well. An audio recording was made of each session after informing the clients that discussions would be recorded. The 3 session recordings were translated to English by a professional translator and verified by one investigator (MKL). Additionally, a video recording was made of one session for archival purposes.

### Focus group discussion guides

The transcript of the focus group discussion is available in Additional file 1 and is outlined in Table 1. Briefly, the introduction welcomed the participants and instructed them on the purpose of the meeting, the manner in which discussions were to be conducted, and explained that the sessions would be recorded by audio taping. Then the discussion proceeded around questions about food consumption patterns and nutritional intake during pregnancy, as well as the intake of fish. Subsequently, the RUSF-DHA was offered for hedonic assessment and discussion of probable acceptance and use as a dietary supplement in pregnancy.

**Table 1:** Layout of the Interview Guide

1. Introduction	
1.1. Welcome and thank the participants for their participation	
1.2. Introductions of the discussion leader and observers	
1.3. Describe the purpose of the Focus Group and DHA discussion	
1.4. Establishing ground rules for the discussion	
1.5. Informing about session recording by audio only or audio and video	
2. Questions regarding food and nutrition during pregnancy	
2.1. Are there any special foods purchased or prepared especially for pregnant women?	
2.2. Do pregnant women need to take vitamins?	
2.2.1. Why or why not?	
2.3. How many of you are taking vitamins while you are pregnant?	
2.4. Where do you get them? Do you pay for them or are they free?	
2.5. Are there any foods that women should not eat when they are pregnant? Why?	
2.6. What should women eat to stay healthy when they are breastfeeding?	
3. Questions regarding fish intake	
3.1. Do you eat Kapenta and fish?	
3.2. How often do you buy Kapenta and fish in your home?	
3.3. How often do you eat it in a week?	
3.4. What type of fish and Kapenta do you eat?	
4. Questions regarding RUSF (asked while smelling and tasting the product).	
4.1. Do you have any allergies to the ingredients in the packet?	
4.2. How does it taste?	
4.3. Do you think other pregnant and breastfeeding women would like it?	
4.3.1. Why or why not?	
4.4. How often would a pregnant or breastfeeding mother eat a product like this?	
4.5. How do you think you might eat this product?	
4.6. What foods might you eat it with or mix it with?	
4.7. What do you like about the product?	
4.8. Any problems or disadvantages with this product?	
4.9. What could be changed about it?	
4.10. Overall, do you like this product?	
5. Please fill out the questionnaire (i.e. hedonic survey) about the product.	

### Analysis of Focus Group Responses.

The transcripts from the 3 focus group sessions were assessed independently by 3 of the study investigators using content analysis methods [20]. The coding units were established a priori for the quantitative data and the script of the sessions was based on the various categories developed into questions as listed in Table 1. Coding centered on 4 themes: knowledge of nutritional needs in pregnancy; knowledge of fish intake for omega 3 DHA consumption; familiarity with RUSF; acceptability and palatability of new RUSF-DHA. After the 3 analysts rated the responses independently, a summative response database was generated and then employed to yield Table 2.

**Table 2:** Summary of focus group responses from transcripts

Question	Responses			
	FG1 (*n* = 7)	FG2 (n = 10)	FG3 (*n* = 10)	Overall
Questions regarding food and nutrition during pregnancy				
Are there any special foods purchased or prepared especially for pregnant women?	Fruits and vegetables; calcium products, milk, yogurt, water, liver	Foods rich in vitamins; fresh milk, kapenta, vegetables, sour milk/yoghurt and fruits, juice, peanut butter and ground nuts	Gorgis protamine, *Nshima*, beans and *Kapenta* or fish, one fruit a day, milk and juice for vitamins, vegetables and peanuts	Calcium products, milk, water, liver, *Kapenta* or fish, vegetables, sour milk, yoghurt, fruits, juice, peanut butter and ground nuts, gorgis protamine, nshima.
Do pregnant women need to take vitamins?	Yes	Yes	Yes	All responded yes
Why or why not?	So that a child can grow healthy	To help have vitamins in the body; to help those who do not eat much food; there is no need to take since we already have a good appetite for food	There are vitamin supplements in form of tablets that are sold in chemistry’s and one is mother care which contains vitamin C, vitamin B12 and other vitamins	So that the body has vitamins and the child can grow healthy. To help those who do not each much food. A connection was made between dietary intake and need for vitamins.
How many of you are taking vitamins while you are pregnant?	At least two participants	At least 4 participants; one respondent said none if good appetite for food	Yes, given in clinic red and yellow supplements;	Most but not all respondents are taking vitamins regularly
Where do you get them? Do you pay for them or are they free?	We are given them in clinic when pregnant	We are given them	Given them	Given to them at the antenatal clinic
Are there any foods that women should not eat when they are pregnant? Why?	Foods high in fat, or fried foods	Not supposed to drink alcohol, smoke cigarettes, or eat dirty clay soil or cold/old *Nshima* (*Chimbala*) because eating *Nshima* from previous day will cause there to be complications during delivery and cause you to soil yourself during delivery.	Alcohol and coke, carbonated drinks and okra or plants in the mallow family because soda is added when cooking it. Mould/mound built by termites because the child will be born with a hardened stomach	Foods high in fat or fried, or old *Nshima*. Should not drink alcohol or coke and other carbonated drinks.
What should women eat to stay healthy when they are breastfeeding?	Proteins like fish and beans and fluids, vitamins, fruits	*Nshima*, vegetables, traditionally brewed drink (*umukoyo*) which helps with having lots of breastmilk; a balanced diet like starch, proteins, vegetables, and something with lots of iron so that the child will have enough blood; vegetables mixed with groundnuts, fruits, vegetables and sour milk, black tea and milk	Foods with proteins, carbohydrates, and vitamins like *Nshima* with beans and vegetables with local fruits or juice.	Proteins like fish and beans, fluids, vitamins, fruits, *Nshima*, vegetables, groundnuts, sour milk, black tea and milk.
Questions regarding fish intake				
Do you eat *Kapenta* and fish?	8/9 yes; 1/9 no *Kapenta,* only fresh fish	6 yes; 1 no fish	Yes, all	Only one respondent did not eat fish at all.
How often do you buy *Kapenta* and fish for your home?	Not queried	Not queried	2 times per wk. (4 participants); every day; 1 per month; twice per month; every day	
How often do you eat it in a week?	2-3times; rarely; 2–3 times; once; every day; not at all	2 times; 3 times; none; 1 time; one told to stop due to gas;	2-3times; rarely; 2–3 times; once; every day; not at all	Anywhere from daily to once a month.
What kinds of fish do you eat?	Fresh bream; buka;	Bream; buka; *Siavonga Kapenta; Mpulinga Kapenta;*	Buka; makerel; bream	Most eat Buka and Bream; some *Kapenta*
What kinds of *Kapenta* do you eat?	*Mpulungu* not *Siavonga*; any big or small	*Mpulungu* and *Siavonga;*	*Mpulungu* and *Siavonga; Chisese* (small fish)	Most eat *Mpulungu* and *siavonga*
Questions regarding RUSF (asked while smelling and tasting the product)				
Do you have any allergies to the ingredients in the packet?	No direct answer. One respondent limits soy products another reacts to *Kapenta* (i.e. intestinal gas)	None	None	Allergies not a problem
How does it taste?	Too much fat, excess sugar or salt	The taste is just okay	Taste of fish oil which is a put off, like the sweetness, bad aftertaste, just fine	Taste was not preferred; some remarked on the fish taste; another on salt and sweetness
How is the after taste?	After taste of peanut butter and the texture is just fine; Okay but the smell isn’t okay	After taste for *Kapenta*, milk and sugar; taste is okay; just okay		The aftertaste was similar to *Kapenta*. Most felt the taste was just fine, while a few suggested that the after taste was similar to peanut butter.
Do you think other pregnant and breastfeeding women would like it?	Not everyone; some will like it, others not depending on the stage of pregnancy; pregnant women can be turned off by smell	Some would like it because it’s good for the child’s health; No, they would not; the ones who are breastfeeding would like the product more than the pregnant mother because pregnant mothers become too choosy with food	Those breastfeeding can like the product because of the omega 3; will be challenging because of the smell; will not like it because they respond differently to tastes, can like it if they remove the fish smell	Most agree the product would be acceptable if it is for the good of the child but the smell could be a problem especially for pregnant mothers who react to some foods especially smelly foods
Why or why not?	Oil, sugar and smell in the product might make them vomit	Those who don’t eat and don’t like the smell of fish cannot manage to eat because it has an aftertaste of fish		Fish smell and aftertaste is not great, may not agree with mothers.
How often would a pregnant or breastfeeding mother eat a product like this?	With this bad smell and texture, I can only manage to eat once a week; twice in a week due to the bad smell; twice in the week because of the needed ingredients in there; just once because its important; if improved and of the same size one can take every day; if it’s this size I can take everyday; I can take once a week; just once a week but if put on other food like bread I think I can eat everyday	They can eat but it must be taken with some other food like bread; the taste is just fine but just the bad smell; it just depends with an individual; some have no problem others have a problem with the smell	Three times a day without the fish smell; even daily without fish smell	Most responded once per week because of the smell; but the general consensus discussion was that if spread on bread or added to another food like rice, it could be eaten more often.
How do you think you might eat this product?		As is or with food	As is or with other food	As is or with other foods like rice or bread
What foods might you eat it with or mix it with?	Bread, tea, warm water or milk, rice	On bread, in porridge or with tea, on rice, frozen	On bread, with rice	On bread, with rice or porridge.
What do you like about the product?	Texture, packaging, I like that it helps the baby, I like it because it has different combinations of nutrients and am sure will get used to it as we keep on eating the product.	All the nutrients are there; aftertaste of *Kapenta*, milk and sugar	It helps the child with development of the brain and good sight but its just the bad smell	Like that it helps the development of the baby, and is nutritious. Easy to carry and many nutrients in one package.
Any problems or disadvantages with this product?	There is too much fat	Smell is bad, tastes like uncooked fish or *Kapenta*, don’t like the milk in it, bad combination of soy and fish	Prefer if it was packed in a tin so that a spoon can be used when eating; with the taste you can’t eat much because of the oil; difficult for those who are allergic to fish	Smell of the product and the oil in it is not great.
What could be changed about it?	Smell		The peanut taste could be stronger than the fish taste, reduce the fish smell and oil	Smell and fish taste.
Overall, do you like this product?	Yes, I like it because it’s important for the child’s health which is critical; It’s okay	I don’t like it; I like it; can eat it with bread; don’t like it but the nutrients will make me take it	Good except for the oil taste, good for it will help babies, product is just okay, think we can get used and eat daily since we have gotten used to folic acid which is not nice as well, will be forcing ourselves to eat.	Do not particularly like it, but will eat it because it is beneficial to the child.

To assess the acceptability of the RUSF-DHA, each participant was offered a 50 g sachet to taste the product and asked to verbally express their response for aroma, texture, flavor, aftertaste, and overall attributes. Each participant also recorded their response on a 9-point hedonic testing instrument. The data were assessed using least squares means.

### Formulation and analysis of the RUSF-DHA

The Ready to Use Supplemental Food with DHA (RUSF-DHA) formulation was based on World Health Organization guidance [8]. The base was peanut butter and supplements were formulated as in iLiNS [21]. A dosage of 400 mg DHA in 50 g RUSF was selected, based on previous studies that recommended this level of DHA to maximize birth outcomes [22]. To supply this level of DHA, the RUSF was supplemented with 5.22% Meg-3™ ‘30’ n-3 Food Oil, a highly refined tuna oil that has a minimum of 9% EPA and 12.5% DHA (DSM Nutritional Products, Inc.). The formulation was prepared in the factory of Edesia Nutrition, Inc. (Kindstown, RI) and transported to Zambia by express shipping.

Each batch of RUSF-DHA was evaluated for fatty acid content after Folch extraction, acid hydrolysis and conversion to methyl esters using gas chromatography and mass spectrometry as is standard in the DiRusso laboratory [23]. A commercial thiobarbituric acid reactive substances (TBARS) analysis kit was used to measure lipid peroxidation (Sigma-Aldrich, St. Louis, MO, USA) of the RUSF-DHA product.

## Results

### Focus group assessment regarding dietary practices and knowledge of nutrition

Participants were enrolled during antenatal clinic visits for sessions being held that day. In total, 3 sessions were convened. Twenty seven women, 7 in the first session, and 10 in sessions two and three, participated in the focus group and subsequent RUSF-DHA taste test. All participants were between 18 and 41 years of age, 16 to 34 weeks of gestation, and 18 to 25 kg/m^2^ of body mass index. Approximately 50% of the women had completed secondary schooling, while 18% had completed only primary school. The remaining participants had some tertiary education and training. An outline and summary of the Interview Guide procedures and questions for the discussions are presented in Table 1 and the complete focus session transcript appears in Additional file 1.

The knowledge of the participants regarding nutritional needs during pregnancy was assessed by querying information about what special foods and supplements including vitamins should or should not be ingested during pregnancy. There was a general consensus in each focus group session that non-fatty foods, dairy products as a source of calcium, and foods containing iron should be consumed on a daily basis (Table 2). The foods cited as containing these nutrients included milk, yogurt, liver, fish or *kapenta*. Other foods noted that should be eaten during pregnancy included fruits and vegetables, ground nuts, beans and *nshima*, a corn-meal based porridge that is a staple of the local diet.

All participants acknowledged that vitamin supplements are recommended during pregnancy for the health of the child, and further reported that these were provided at no cost at the antenatal clinic or might be purchased at a pharmacy. Referring to the free vitamin supplements, one participant commented that,

“*Yes, always when someone is pregnant they are given vitamins although we can’t know what they do with them at home whether they throw or they take them*”. *F01-R1*

There was a general acknowledgement that vitamins are particularly important when food is limited on a daily basis. Specific vitamins mentioned included vitamin C and B12. A need for calcium and iron supplementation was mentioned in two of the sessions. However, despite the access and knowledge about vitamins, not every woman was taking them daily. A respondent stated,

“ … *there is no need to take since we already have a good appetite for food*”. *F02-R1*

Participants indicated that some foods and beverages should be avoided, including those that are high in salt and fat, soft drinks, and alcohol. Smoking was also discussed for its negative health impacts. One participant commented that,

“*she can eat anything unless if its causing her to have nausea.*” *F01-R2*

This comment initiated a discussion of the negative impact on appetite of strong odors and flavors during pregnancy. There was also a discussion in one group about the practice of eating dirt or clay, a practice that is common in some cultures [24–26]. The participants acknowledged that this practice was discouraged by healthcare workers.

### Assessment of fish consumption and knowledge of health benefits of fish

Certain fish species are a natural nutritional source of DHA [7, 27]. Therefore, fish intake was extensively queried and discussed. Those participants that ate fish did so on average 2–3 times per week; one participant said she eats it every day and one did not eat fish at all. Fish was acknowledged as being a good nutritional staple during pregnancy, but not specifically as related to brain or cognitive development of the fetus or infant. That information was provided by the focus group facilitators as part of the introduction describing the purpose of the focus group and importance of DHA intake for infant development (Additional file 1). The common fish species purchased and consumed were: bream made up of common types of *cichlidae* similar to tilapia; *mpulungu buka* (*Lates stappersi*); *kapenta*, a type of fresh water sardine (*Limnothrissa miodon* and *Stolothrissa tanganicae*); and *chisense* (*Neobola nweruensis*). One participant mentioned eating mackerel. Of these, *kapenta*, *chisense* and mackerel contain significant amounts of DHA (10% or more of the total fatty acids) [7, 27]. *Kapenta* intake was specifically queried in the survey for this reason. Intake varied from none to 2–3 times per week. It should be noted most respondents who ate fish generally mentioned bream and *buka,* which are not significant sources of DHA [27, 28].

### RUSF formulation

A major goal of this work was to develop and assess an RUSF to contain fish oil as a method to deliver macronutrient and micronutrient supplements and DHA to women at risk for food insecurity and malnutrition during pregnancy and lactation since this is a concern in Zambia [5, 29]. The lipid based nutrient supplements are deemed a valid delivery vehicle for DHA as the oils are miscible in the nut paste, low in water, as well as air and light tight, which helps to maintain the integrity of the highly unsaturated fatty acids to prevent or reduce oxidation [30]. The base of the RUSF-DHA developed for this study was peanut butter, supplemented with non-fat milk and whey powders, sugar, and mixed natural tocopherols as preservatives with a vegetable oil mixture as listed in Table 3. Additional micronutrients, including minerals and vitamins, were added as recommended [8]. Preliminary formulation tested DHA levels at 300, 600 or 900 mg per 20, 50 or 75 g of RUSF. We sought the highest dose palatable, based on aroma and taste in small group tastings at Edesia, Inc. and UNL. The 300–600 mg doses were equally accepted, while the 900 mg dose was rated as having the strongest “fishy” odor and aftertaste. The final formulation of approximately 400 mg DHA per 50 g supplement was selected to reach levels informed by studies in the US and elsewhere as suitable for optimal birth and infant developmental outcomes [14, 22, 31–35].

**Table 3:** Composition of the RUSF-DHA (50 g serving)

Ingredients	Contents	
	Non-hydrogenated vegetable oil (containing soybean oil, canola oil, palm oil), peanuts, nonfat milk powder, sugar, whey powder, Meg-3™ ‘30’ n-3 INF Oil (refined fish oil from tuna), identity preserved mixed natural tocopherols, ascorbyl palmitate, stabilizer (hydrogenated vegetable fat), and vitamin and mineral complex.	
	Amount/50 g	kcal/50 g
Fat	20.4 g	184
Protein	6.8 g	27
Carbohydrate	21.4	86
Calories		297
Meg-3™ ‘30’ n-3 INF Oil	5.22% (400 mg DHA)	
Iron	22 mg	
Vitamin C	90 mg	
Folic Acid	600 mcg	
Iodine	330 mcg	
Water Activity	0.367	
Enterobacteriaceae	< 10 cfu/10 g	
Salmonella	0 cfu/375 g	
Total Aflatoxin	< 0.2 ppb	

RUSF-DHA formulation by Edesia Nutrition, Inc., Kingstown, RI. Note, not all micronutrients listed

**Fig. 1:**
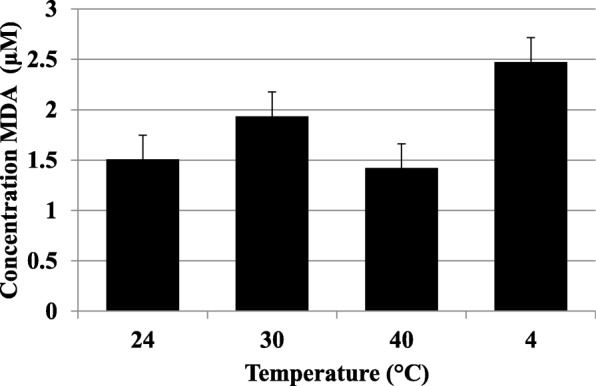
Estimation of lipid peroxidation after storage. RUSF-DHA samples were stored at various temperatures for 6.5 months. There is no significant difference between samples (One way ANOVA (*p* = 0.69; JMP Pro v12))

Fatty acid analysis of the final product indicated about half of the fat was monounsaturated, primarily oleic acid and about 13.5% was the saturated fatty acid palmitate (Table 4). The polyunsaturated fatty acids (PUFA) (linoleate and linolenate) comprised about 50% of the total fatty acids and the long chain highly unsaturated PUFA were 3% total. As expected of the latter, arachidonic acid levels were low at 0.24 mol% since an enriched source of this long chain PUFA was not added, while EPA and DHA were 0.57 and 2.3 mol% total FA, respectively, from the fish oil. Since oxidation of DHA and other unsaturated fatty acids could lead to reduced palatability and unfavorable odors (i.e. “fishiness”), a commercial assay was performed, which demonstrated no significant increase in oxidized lipids over 6.5 months storage with temperature (Fig. 1).

**Table 4:** Fatty acid analysis of RUSF-DHA

			mg/50 g Serving (SEM)^1^		
*Fatty Acid*		RT (T_o_)	RT (T_6.5_)	30 °C (T_6.5_)	40 °C (T_6.5_)
Myristic	C14:0	74.2 (2.71)	77.06 (2.55)	75.13 (1.50)	73.77 (1.34)
Palmitic	C16:0	1762 (34.92)	1860.6 (28.97)	1716.44 (7.96)	1708.17 (17.2)
Palmitoleic	C16:1	94.73 (2.32)	100.30 (0.53)	96.59 (2.47)	97.31 (1.53)
Stearic	C18:0	771.07 (14.09)	810.53 (11.99)	763.54 (2.78)	756.51 (3.85)
Oleic	C18:1	7032.46 (172.12)	7535.15 (83.29)	6895.01 (124.53)	6870.18 (130.09)
Linoleic	C18:2 n-6	2490.37 (52.82)	2639.32 (26.96)	2510.33 (54.07)	2523.96 (37.58)
Linolenic	C18:3 n-3	787.94 (16.77)	827.30 (9.78)	806.42 (21.66)	816.78 (9.97)
Arachidonic	C20:4 n-6	36.89 (0.95)	36.9 (1.03)	36.43 (1.80)	35.57 (0.29)
Eicospentaenoic	C20:5 n-3	84.34 (2.61)	91.1 (1.37)	87.43 (2.20)	88.51 (1.17)
Docosahexaenoic	C22:6 n-3	375.52 (11.79)	386.50 (11.69)	402.38 (10.70)	405.17 (5.83)
			Relative mol%		
SFA	C14 + C16 + C18	19.61 (0.08)	19.44 (0.07)	19.36 (0.24)	19.26 (0.14)
MUFA	C16:1 + C18:1	50.1 (0.2)	50.49 (0.06)	49.49 (0.18)	49.35 (0.19)
PUFA	C18:2 + C18:3	23.21 (0.07)	23.09 (0.04)	23.64 (0.17)	23.83 (0.03)
HUFA	C20:4 + C20:5 + C22:6	3.08 (0.04)	3 (0.05)	3.28 (0.04)	3.3 (0.02)
n-6:n-3		2.11 (0.01)	2.14 (0.01)	2.05 (0.02)	2.04 (0.04)

^**1**^RT, room temperature; 6.5 indicates the number of months in storage at the indicated temperature. SFA, saturated fatty acid; MUFA, monounsaturated fatty acid; PUFA, polyunsaturated fatty acid; HUFA, highly unsaturated fatty acid

### Comments on the RUSF-DHA while sampling the food

After the discussion of diet and nutrition information, the participants were offered a 50 g sachet of the RUSF-DHA to taste, smell, and sample. Impressions of the food product were recorded and transcribed from the discussions (Table 2) as well as recorded in a standardized hedonic test (Table 5). None of the participants had any noted allergies to the product ingredients, although one indicated a problem with intestinal gas after eating dried fish and another said she generally limits soy products. The most negative remarks revolved around the aroma, which was felt to be too “fishy”. For example, remarks included:

“*the taste is just fine only the smell is what I don’t like*”. *F03-R5*

Most participants felt the taste was acceptable, although a few thought the taste was salty and sweet, which is a common response of adults to lipid-based RUTFs. The texture was acceptable to all.

**Table 5:** Summary of focus group responses to sensory test (27 participants)

Attribute	Hedonic Scale* Mean (Range)	Oral Comments	Written Comments
Aroma	4.41 (1–9)	Don’t like the fish smell	Aroma needs to be changed
		Bad smell	Smell is unfavorable
			Aroma is not very appealing
			There is a fish smell
			Aroma should be changed
Texture	8.30 (4–9)	It’s okay, smooth and thick	Texture is OK
			Texture is good
Flavor	7.07 (2–9)	Taste is just fine	Flavor is OK
		If the peanut butter taste were stronger than the fish it could be much better	Flavor is moderately nice
			Like all of the flavors
Aftertaste	4.11 (1–9)	Bad aftertaste of fish	Aftertaste is OK
		Peanut butter aftertaste	Aftertaste is unfavorable
			Aftertaste is not very nice
Overall	7.11 (1–9)	Pregnant and breastfeeding mothers can take it because it’s good for the child’s health	Sugar level is too much
		Would eat daily without the fish smell	I like the product and everything is okay
		It is good because it helps the child with development of the brain and good sight	They are all fine. It has the benefit for my baby.
			The product is okay
			It has got all the vitamins we need
			The sample is generally good as it seems to contain all nutrients
Likes overall	7.64 (6–9)	The peanut butter taste	Like it because it has good advantages
		The product helps the child	The product is okay if the fish smell can be reduced
Dislikes overall	1 (1 respondent)	Fishy/oily taste and smell	Aftertaste is not great
			Smell is fishy
			Texture is too thick

Least squares means for the aroma, texture, flavor, aftertaste, and overall attributes of the RUSF using a 9-point hedonic scale, *where 9 = Like Extremely, 8 = Like Very Much, 7 = Like Moderately, 6 = Like Slightly, 5 = Neither Like nor Dislike, 4 = Dislike Slightly, 3 = Dislike Moderately, 2 = Dislike Very Much and 1 = Dislike Extremely. Attributes were assessed by focus group participants

The participants were asked how often they thought a pregnant or breastfeeding mother would eat this product. The answers varied from only once per week, because of the smell, to three times a day if the fish odor could be limited. Most participants agreed that the RUSF-DHA would be more likely to be acceptable for every day intake if mixed with another food such as rice or eaten as a spread on bread.

While many participants expressed some concern about the smell and flavor, it was generally agreed that the health benefits to the developing child would take precedence over taste and smell to drive the individuals toward acceptance. In hedonic testing, the flavor actually rated quite high at 7/9, while the aftertaste was rated as moderate at 4/9 on a scale of 9 (like extremely) to 1(dislike extremely) (Table 4). Generally, the participants agreed that if the RUSF-DHA provided nutritional benefit to the child’s development, then it would be acceptable to pregnant and lactating women. One stated,

“*I like it because it’s important for the child’s health which is critical, mothers should take it*”, *F01-R6*

and another,

“*I don’t like it but just the nutrients will make me take it*.” *F02-R6*

Additionally, it was mentioned that the smell might be less objectionable during lactation, rather than during pregnancy when certain foods and odors can initiate nausea:

“*I think the ones who are breastfeeding can like the product more than the pregnant mothers because pregnant mothers become too choosey with food*.” *F02-R3*

## Discussion

In the present work, we interrogated knowledge about nutrition during pregnancy and eating habits of pregnant women in an antenatal clinic population in Zambia where there are high rates of food insecurity, malnutrition, and stunting. We also developed and evaluated acceptability of a lipid based-RUSF formulation that contained fish oil to provide a source of DHA. The RUSF-DHA was designed for use by malnourished women during the first 1000 days, from conception through 24 months, during the critical period of brain and neuronal development [14]. The DHA dosage was based on studies in the U.S. and elsewhere that were aimed at evaluating DHA for improved birth, cognitive, and developmental outcomes for women and their infants [15, 34, 36–38].

During the focus group sessions, participants generally expressed knowledge that certain food groups are required for the health of the developing child, including vegetables, fruit, milk and dairy products, beans, corn meal porridge, meat, and fish. The responses reflected the fact that the importance of a nutrient-rich diet during pregnancy was generally acknowledged. Nutrition counseling is routinely incorporated as part of the prenatal program offered by the UTH ANC in Lusaka. Thus, the women were receptive of a relatively new concept that certain fats, namely DHA, were also important for brain development of the child before and after birth. In fact, the value of the nutrient, DHA, drove the acceptability of the RUSF, even though the sensory testing revealed a dislike for the smell of the food and some aspects of the taste including an aftertaste.

Since the supplement should be consumed daily over months during pregnancy and lactation, we also sought to determine acceptability in a population at high risk for food insecurity. The final formulation contained approximately 400 mg DHA per 50 g serving. The most common criticisms of the product were that it had a fishy smell and taste. Despite this finding, the product was found to be acceptable because of its potential value to healthy birth and developmental outcomes for the child. Ultimately, the development of the child took precedence over the mothers’ tastes. It was generally agreed that the 50 g supplement would be consumed for this reason, especially if added to another component of a standard diet such as rice or bread.

It is noteworthy to mention that, while the RUSF-DHA product was designed for use by individuals at risk for chronic malnutrition, the participants in this study were not clinically malnourished as all fell within the range of body-mass index (BMI) between 18 and 24 pre-pregnancy. However, the need for the product by this clinic population overall is justified as it was determined during enrollment that within the general client base of the clinic, 23% (168/735) of women visiting for the first prenatal screening were clinically malnourished with a pre-pregnancy BMI ≤ 16. The national population average in Zambia for malnutrition is 17%, thus these malnourished women were excluded from the focus group panels to meet the inclusion criteria. However, the malnourished population is the ultimate target for the RUSF being developed to provide one supplement that will meet all the various micronutrient needs of women during pregnancy and lactation [24]. Additional studies are underway in Zambia to assess the DHA status of pregnant women and to examine the possible additional consequences of infectious disease including Human Immunodeficiency Virus (HIV) where prevalence is 22% of pregnant women [1].

Both women and children diagnosed with severe or moderate malnutrition have positive outcomes when provided Ready to Use Therapeutic or Supplemental Foods (RUTF and RUSF, respectively) as part of their therapeutic care [8, 12, 24, 39, 40]. Studies surrounding such small quantity lipid based nutritional supplements (LNS) were the focus of the International Lipid-Based Nutrient Supplements Project (iLiNS), which sought to evaluate a wide variety of impacts using various formulations of small quantity LNS (i.e. 20 g total daily) [11]. Encouraging outcomes of some these trials included, for example, increased birth size and length of infants of primiparous women in Ghana taking a small quantity (20 g) RUSF daily from 20 weeks gestation until delivery [21, 41, 42]. Thus, RUSF are often recommended in chronic malnutrition to add essential nutrients to the standard diet during food insecurity. Supplemental feeding projects like the completed Rainbow Project in Zambia further provide evidence for the value of RUTF use [43]. Overall, in the present study the participants displayed a general knowledge of the types of food that are recommended for consumption during pregnancy to provide adequate nutrient intake for mothers and infants. Some of this nutritional advice had been provided by the staff of the ANC during clinic visits. While eating fish to provide specific nutrients like DHA was not necessarily a component of the participants’ prior knowledge base, fish are part of the standard diet for the population and are eaten by most 2–3 times per week. However, the type of fish purchased and consumed was variable and some commonly mentioned types including bream and *buka* that are not a good source of DHA [7, 27].

Common sources of high DHA oils include certain species of fish (e.g. salmon, tuna, sardines) and/or algae [44–46]. In Western cultures, dietary DHA levels are generally very low and dietary supplementation for pregnant and lactating women and infants is highly recommended [14, 16, 47]. No studies have evaluated RUTF or RUSF supplemented with a DHA containing oil and only one has evaluated the combination of RUTF and fish oil capsules in a clinical trial setting [48]. Therefore, it remains controversial as to whether or not there is value in adding DHA and/or limiting the ratio of n-6 to n-3 fatty acids to improve health outcomes with these food supplements. Additionally, not all food insecure populations may require DHA supplementation. For example, if the traditional diet includes fish high in DHA as a dietary staple, as is true in Malawi, such supplementation would be unnecessary [49]. Therefore, it will be important to test the need for the product based on assessment of the omega 3 index in blood in the underweight and malnourished population of Zambian women. If low, then supplementation with the RUSF-DHA for outcomes related to fetal and infant development, as well as birth outcomes, must also be evaluated in future studies.

There were limitations to this focus group study, including the small numbers of participants. The size of the groups was kept low at 7–9 individuals to encourage conversation and participation by all members of the group. The final number at 27 individuals followed recommendations for this type of survey of climate and knowledge surrounding dietary behaviors and knowledge of nutrition [19]. The room chosen for the discussion sessions was also suboptimal as non-participants could not be excluded from the room. The population of participants was limited to the greater urban area of Lusaka. Additional focus groups are planned for rural locations in the future. Language may also have been a barrier to some individuals as the introduction and discussion questions had to be translated by the group facilitators to accommodate all participants. Despite these limitations, this study provided useful insight into the beliefs and practices of nutrition in pregnancy among ANC UTH clients.

## Conclusions

The participants expressed willingness to accept new information regarding the need for essential fatty acids, particularly DHA, for the health of their infants during pregnancy and lactation. It was apparent that additional knowledge regarding the benefits of fish as a source of protein, vitamins, and essential fatty acids is needed as part of the nutrition and clinical services provided. Specifically, there should be education about including those types of fish that are high in DHA such as *kapenta* in the standard diet. As mentioned above, it is also critical to conduct additional studies to evaluate DHA supplementation as the RUSF-DHA for food insecure and malnourished women in pregnancy and lactation. For the well-nourished population, increased levels of DHA intake should be encouraged by counseling to eat *kapenta* and other high DHA-containing fish.

## Supplementary information

**Additional file 1.** The complete script of the focus group interviews.

## Data Availability

The datasets generated and analyzed during the current study are not publicly available since participants did not give consent to publicly sharing their information, but summaries of the information are either included in the manuscript or available from the corresponding author on reasonable request.

## Abbreviations

AA: arachidonic acid
ANC: antenatal clinic
BMI: Body Mass Index
DHA: docosahexaenoic acid
EPA: eicosapentaenoic acid
HIV: Human Immunodeficiency Virus
RUSF: Ready to use Supplemental Foods
RUTF: Ready to Use therapeutic Foods
