# Supplementary material for: Healthy pregnancies and essential fats: focus group discussions with Zambian women on dietary need and acceptability of a novel RUSF containing fish oil DHA
Source: BMC Pregnancy Childbirth. 2020 Feb 10;20:93. doi: 10.1186/s12884-020-2783-8 (PMC7011535; doi:10.1186/s12884-020-2783-8)
Supplement: Supplementary file 1 — Additional file 1. The complete script of the focus group interviews. [file 12884_2020_2783_MOESM1_ESM.docx]

Focus Group Evaluation of Enriched RUSF

STUDY ID: _ Date: _

FOCUS GROUP INTRODUCTION

*WELCOME. Thanks for agreeing to be part of the focus group. We appreciate your willingness to participate.*

*INTRODUCTIONS : Moderator; Assistant Moderator: My name is* .............

*PURPOSE OF FOCUS GROUPS:/ would like to ask you some questions today to learn about common nutrition practices in your community and to receive your feedback on a new nutritional product for pregnant and lactating women.*

*GROUND RULES*

1. *WE WANT YOU TO DO THE TALKING.*

*I would like everyone to participate. I am simply interested in understanding your opinions and thoughts. You are not required to answer any of the questions if you do not feel comfortable doing so. I need your input and want you to share your honest and open thoughts with us.*

*I may call on you if I haven't heard from you in a while.*

1. *THERE ARE NO RIGHT OR WRONG ANSWERS*

*Every person's experiences and opinions are important. Speak up whether you agree or disagree. We want to hear a wide range of opinions.*

1. *WHAT IS SAID IN THIS ROOM STAYS HERE*

*We want folks to feel comfortable sharing when sensitive issues come up.*

1. *WE WILL BE TAPE RECORDING THE GROUP*

*We want to capture everything you have to say. We don't identify anyone by name in our report. You will remain anonymous.*

*Does anyone have any questions before we get started?*

***The first topic I'd like to talk to you about is how women can stay healthy when they are pregnant.***

- 1. Are there any special foods purchased or prepared especially for pregnant women?
  2. Do pregnant women need to take vitamins (or medicines)?
     1. Why or why not?
  3. How many of you took (or are taking) vitamins while you were (are) pregnant?
     1. If yes, where do you get them? Do you pay for them or are they free?
  4. Are there any foods that women should NOT eat when they are pregnant? Why?
  5. What should women eat to stay healthy when they are breastfeeding?
  6. Do you take Kapenta and Fish?
  7. How often do you buy Kapenta and Fish in your home?
  8. How often do you eat it in a week?
  9. What type of FISH and KAPENTA do you eat?

***I'd now like to show you a new product we are working on developing for women who are pregnant and breastfeeding just like you****. First I will show it to you, and then I will give you* ***some more information about it. (Show packaging and product).***

1. What do you think this product is?
2. Is it similar to anything you've seen before?

# Now I will give you a chance to taste this food (if you'd like to) and tell me what you think about it. (Pass around packets and allow all participants to taste. It is made of peanuts, oil, sugar, milk, and soy and it is fortified with vitamins that women need to stay healthy when they are pregnant and breastfeeding. It can be eaten directly from the sachet and does not need to be mixed with water or kept refrigerated. Explain that the product contains peanuts, dairy, and soy, and anyone with allergies should not eat it.)

1. What do you think of the taste?
2. What do you think of the texture?
3. How often per week would a woman eat a product like this?
4. How do you think you might eat this product?
5. What foods might you eat it with or mix it with?
6. Would eating it directly from the sachet be acceptable?
7. What do you think are the good things about this product?
8. What do you think might be problems or disadvantages of this product?
9. Do you think other pregnant and breastfeeding women would like it?
   1. Why or
   2. Why not?
10. Overall, do you like this product?
11. Would you consume it daily throughout pregnancy and breastfeeding?

You will be asked to smell the sample and then taste it. Record how you like the smell and taste by answering the questions below by filling in the circle of the response that best describes your like or dislike of the supplement
